# Rationale and study protocol for a randomized controlled trial to determine the effectiveness of a culturally relevant, stress management enhanced behavioral weight loss intervention on weight loss outcomes of black women

**DOI:** 10.1186/s12889-022-12519-z

**Published:** 2022-01-28

**Authors:** Acadia W. Buro, Monica Baskin, Darci Miller, Tayler Ward, Delia Smith West, L. Robert Gore, Clement K. Gwede, Elissa Epel, Tiffany L. Carson

**Affiliations:** 1 Department of Health Outcomes and Behavior, Moffitt Cancer Center, 4117 E Fowler Ave, Tampa, FL 33617 USA; 2 Division of Preventive Medicine, University of Alabama at Birmingham, Birmingham, AL USA; 3 Department of Exercise Science, Arnold School of Public Health, University of South Carolina, Columbia, SC USA; 4 Department of Biostatistics and Bioinformatics, Moffitt Cancer Center, Tampa, FL USA; 5 Department of Psychiatry, University of California San Francisco, San Francisco, CA USA

**Keywords:** Obesity, Cancer prevention, Intervention, Randomized controlled trial, Chronic stress, Black women

## Abstract

**Background:**

Obesity is a persistent public health concern and a risk factor for many chronic diseases including at least 13 different cancers. Adult Black females have the highest prevalence of obesity (57%) compared to other racial/gender groups in the U.S. Although behavioral weight loss (BWL) interventions have demonstrated effectiveness, Black females tend to lose less weight than White counterparts. The higher prevalence of chronic psychological stress reported by Black females may contribute to their disproportionate prevalence of obesity and observed suboptimal weight loss. This study will examine the effectiveness of a 12-month culturally-targeted, stress management-enhanced BWL intervention on weight loss and stress reduction among Black females in a fully-powered randomized, controlled trial.

**Methods:**

Adult Black females with obesity (*n* = 340) will be randomized to either a culturally targeted stress management-enhanced BWL intervention (BWL-Stress) or the same BWL intervention alone (BWL-alone). The primary outcome is weight change at month 6. Secondary outcomes will include changes in stress measures (e.g., perceived stress, cortisol), energy intake, and physical activity at month 6. We will also assess process measures (e.g., treatment adherence, treatment burden). Each outcome will also be evaluated at month 12 to assess longer-term effects of the intervention.

**Discussion:**

This novel approach for enhancing an evidence-based BWL program with culturally-targeted stress management strategies for Black females addresses an understudied barrier to effective weight management among a population at high risk for obesity and obesity-related chronic diseases. This study will potentially elucidate psychological or behavioral mechanisms linking our novel intervention to study outcomes. If the intervention is proven to be effective, this study will have significant clinical and public health implications for weight management among Black females.

**Trial registration:**

This study was registered on ClinicalTrials.gov, identifier NCT04335799t, on April 6, 2020.

## Background

In the US, Black adults are disproportionately affected by cancer, with the highest death rate of any racial or ethnic group [1]. The lifetime risk of cancer for Black adults is 1 in 3, and more cases occur among females than males [2]. Nearly 40% of US adults have obesity, a risk factor for 13 types of cancer [3]. The highest prevalence of obesity is among Black females, at approximately 57% [4]. Given that obesity is a strong risk factor for cancer [5], effective strategies for weight management are needed to combat the obesity epidemic and the resulting risk for several types of cancer and other chronic conditions, especially in those at highest risk.

Although behavioral weight loss (BWL) interventions have been effective at promoting weight loss in some individuals with obesity, these programs still result in less weight loss among Black females compared to White counterparts [6, 7]. Even the Diabetes Prevention Program (DPP) Intensive Lifestyle Intervention, which produced clinically meaningful weight loss (3-5% of baseline weight) among Black women (4.7 kg weight loss at 6 months), achieved 2.8 kg greater weight loss in White women and significantly greater weight loss among all race-gender groups (*p* < 0.01) except for Black men, indicating a need to examine sociocultural and environmental factors for successful BWL interventions among Black women [8]. Disparities in weight loss outcomes of Black and White individuals have also been reported in several other BWL trials [9]. To improve weight loss outcomes among Black women, research on culturally-adapted interventions and mechanistic links between cultural factors and weight loss is warranted [10].

Psychological stress is associated with weight gain over time and higher body mass index (BMI) [11–13]. In previous BWL interventions, individuals with higher stress at the start of the weight loss program went on to lose less weight than those with lower stress [14, 15]. Given that Black females report higher chronic stress than other groups [16], have a unique stress experience due to the intersection of race and sex [17], and tend to employ coping strategies that may be detrimental to health [18–22], the role of stress on weight control efforts may be particularly relevant for Black women. There is a well-established literature on stress reduction interventions that address racial/ethnic, health, and socioeconomic differences in stress exposure and stress-related outcomes [16], as well as observational relationships suggesting that high stress levels may contribute to enduring and increasing adiposity [11]. However, there remains a gap in the literature about whether adding a culturally-targeted stress management component to an evidence-based BWL intervention will produce greater weight loss than the standard intervention. A 2018 review [13] reported that only two small studies have investigated how adding stress management training to BWL programs affects weight loss: one reported significant positive effects among Greek women [23], and the other, conducted in a sample of Black women, reported encouraging trends but was underpowered to detect significance [24]. In addition, a randomized BWL program that tested the addition of mindfulness training for stress reduction in a predominantly (60%) White sample did not lead to greater weight loss, although it led to greater glucose control, a secondary outcome [25]. The participants of color attended fewer sessions in the mindfulness condition, and had less weight loss, again suggesting culturally tailored stress reduction is needed [26]. The purpose of this study is to examine whether a culturally-targeted, stress management-enhanced BWL intervention will improve weight loss and stress reduction among Black females in a fully powered randomized, controlled trial.

### Aims and hypotheses

This study examines whether a culturally-targeted, stress management-enhanced BWL intervention (BWL-Stress) is effective in reducing weight compared to a standard BWL intervention (BWL-Alone) among adult Black females with obesity and elevated stress levels. The primary aim is to conduct a randomized controlled trial to determine the effects of BWL-Stress on body weight compared to BWL-Alone among adult Black females with obesity and elevated perceived stress. Black women with co-existing obesity and elevated stress as measured by a validated survey will be randomized to BWL-Stress or BWL-Alone. We hypothesize that BWL-Stress will lead to greater weight loss at 6 months than BWL-Alone. The second aim is to evaluate the effects of BWL-Stress on multidimensional stress as measured by a validated survey instrument and a stress biomarker. Perceived psychological stress will be measured by the Perceived Stress Scale (PSS-10) [27] and the Stress and Adversity Inventory (STRAIN) [28], and salivary cortisol (assessing the cortisol awakening response) will be collected as an objective measure of stress at baseline and multiple follow-up time points to be able to compare changes by intervention group over time. We hypothesize that BWL-Stress will lead to greater reduction in perceived stress and more improvement in cortisol level at 6 months than BWL-Alone. The third aim is to determine whether the relationship between BWL-Stress and weight change is mediated by perceived stress, energy intake, and/or physical activity. A multiple mediation model will be used to determine if perceived stress level, energy intake, and/or energy expenditure explain observed relationships between the stress management-augmented intervention and weight change. We hypothesize that the relationship between BWL-Stress and 6-month weight change will be partially mediated by PSS-10 score, energy intake, and physical activity.

## Methods/design

Participants (*n* = 340) will be randomized to one of two treatment conditions: BWL-Stress or BWL-Alone. Both groups will receive the same evidence-based BWL program over 26 sessions. In addition to the BWL program, the BWL-Stress group will receive training on stress management, whereas the BWL-Alone group will receive education on general women’s health topics as an equivalent attention control. Participants will meet for group-based sessions weekly for 4 months, bi-weekly for 2 months, and then monthly for 6 months.

### Participants

Black females aged 21-75 years with a BMI of at least 30 kg/m^2^ and elevated stress levels (PSS-10 score ≥ 13) will be eligible for inclusion. Individuals will be excluded if they: 1) are pregnant or are planning to become pregnant in the next year, 2) have a known major medical or psychological condition known to influence body weight (e.g., medicated or poorly controlled diabetes [fasting blood glucose > 126 mg/dL], cardiovascular event in the preceding 12 months, history of gastric bypass surgery, bariatric surgery, or eating disorder), 3) history of psychiatric hospitalization in past 2 years, 4) history of substance abuse or eating disorder, or 5) any condition for which a medical professional has suggested diet modification, physical activity, and/or weight reduction would be contraindicated.

### Sample size and power

While primary analysis will be adjusted for prognostic factors for weight loss, sample size was determined using an equal variance t-test. Preliminary data identified a 1.4 kg loss for Lifestyle alone and 2.7 kg loss for Lifestyle + Stress interventions, with standard deviations of 2.1 and 4.0 kg, respectively, yielding a Cohen’s d of 0.44 [24]. As these measures were taken at 3 months, and the current study is assessing 6-month weight loss, larger effects are likely; however, to reduce the likelihood of using an effect size which is too large [29], the study has been powered for a Cohen’s d of 0.35. This effect comes from assuming the above with both groups having a 4 kg standard deviation. Thus, with 85% power and a Type 1 error rate of 0.05, 148 participants are needed per group for a total of 296 participants. To allow for 10% attrition without loss of power, 340 participants (170 per group) will be enrolled and randomized.

### Recruiting and screening eligible participants

Participants will be recruited from the Tampa Bay area through flyers, word of mouth, and small media (e.g., websites, clinical trials advertisements) using culturally appropriate recruitment materials designed to engage Black women. Established relationships between Moffitt Cancer Center and the Tampa Bay Community Cancer Network will also be leveraged to aid in the recruitment of community volunteers for this research study. Recruitment campaigns will be conducted across Years 2-5 of the project to enroll approximately 85 participants per each of four waves, allowing for delivery of at least four treatment groups per cohort (two BWL-Stress and two BWL-Alone groups) with approximately 21 participants per group. Based on a conservative estimate of a 35% recruitment yield obtained from our preliminary research [24, 30], we anticipate pre-screening approximately 970 individuals (~ 323 per year) to enroll 340 participants over four waves. Method of recruitment will be documented.

Interested individuals will initially be screened by telephone by research staff. Based on preliminary eligibility, individuals will be scheduled for a baseline visit to confirm eligibility and complete the informed consent process. Eligible participants will complete all baseline assessments prior to randomization. The random allocation sequence will be generated with computer-generated random numbers.

### Intervention

Both treatment conditions will include a DPP-based lifestyle change curriculum presented in a group-based format in 26 sessions over a 1-year period. DPP is a structured lifestyle change program focused on reducing caloric intake and increasing physical activity to at least 150 min per week [31]. DPP employs evidence-based behavior change strategies such as self-monitoring and goal setting to promote lifestyle changes. Based on the original DPP study in which participants lost 5-7% of baseline body weight and the risk of developing diabetes was reduced by 58% in high-risk adults [31], as well as follow-up studies showing similar successes [32, 33], DPP has been adopted as a Centers for Disease Control (CDC)-recognized lifestyle change program. Consistent with the CDC-recommended meeting schedule, participants will meet once a week for 4 months (sessions 1 to 16), every other week for 2 months (sessions 17 to 20), and once a month for 6 months (sessions 21 to 26) [34]. Importantly, the two conditions will be equivalent in the format, frequency, and duration of contacts, and will differ only in the material that will supplement the DPP-based behavioral lifestyle change curriculum.

#### Community advisory board

A Community Advisory Board (CAB) consisting of five Black women from the Tampa Bay area contributed personal and professional opinions about stress management curriculum materials. CAB members included two consultants, a director/program manager, a nurse, and a public health manager. All CAB members had experience working in a leadership role in a community nonprofit or business. These women were selected to serve on the CAB primarily because of their community/health advocacy experience, their racial- and sex-concordance with the targeted population for the study, and because they represent different perspectives (e.g., race, sex, age, geography) that our research team wanted to ensure were reflected in the intervention materials. The CAB met for four weekly 1-h sessions, in which stress management session content, impact, and usability, as well as cultural relevance of wording and images, was discussed. Members were sent 6-7 session documents about 5 days prior to each meeting. Each stress management document was reviewed individually, and each CAB member was compensated $500 after all meetings were completed.

### BWL-stress

BWL-Stress will deliver the DPP-based lifestyle change curriculum during 26 60-min group sessions, each of which will also include an additional 20-min training session focused on introducing and reviewing stress management strategies (Table 1). The total session time will be approximately 80 min. Our pilot study demonstrated the feasibility of introducing stress management techniques within a 20-min training [24], and other studies have shown that 15-20-min bouts of focused breathing, mindfulness, and meditation can lead to documented reductions in stress indicators and improved cognition [35, 36]. Participants will be encouraged to apply the stress management strategies as frequently as needed outside of group meetings.

**Table 1:** Curriculum for BWL-Stress and BWL-Alone during each study phase

DPP Lifestyle Change Curriculum	Women’s Health (BWL^**a**^-Alone)	Stress Focus (BWL-Stress)
**Weekly Sessions**		
Session 1: Welcome to DPP	Breast Self-care	Intro to Stress and Health
Session 2: Be a Fat and Calorie Detective	Skin Care	Identifying/Acknowledging Triggers of Stress
Session 3: Three Ways to Eat Less Fat and Fewer Calories	Dental Care	Meditation with Focus on Peace, Spirituality, Pride
Session 4: Healthy Eating	Heart Health	Benefits of Massage
Session 5: Move Those Muscles	Sleep Habits	Values Clarification
Session 6: Being Active – A Way of Life	Alcohol Use	Exercise as a Stress Reliever
Session 7: Tip the Calorie Balance	Tobacco Cessation	Progressive Muscle Relaxation
Session 8: Take Charge of What’s Around You	Domestic Violence	Navigating Misinformation and Finding Quality Resources for Your Needs
Session 9: Problem Solving	Age-Appropriate Cancer Screenings	Control What You Can, Accept What You Can’t
Session 10: Four Keys to Healthy Eating Out	Addictive Behaviors	Expressing Gratitude
Session 11: Talk Back to Negative Thoughts	Health Insurance	Developing an Action Plan with Achievable Goals
Session 12: The Slippery Slope of Lifestyle Change	Vaccines	Challenging dichotomous thinking
**Bi-Weekly Sessions**		
Session 13: Jump Start Your Activity Plan	Navigating Healthcare in the Digital Age	Financial Management
Session 14: Make Social Cues Work For You	Environmental Health	Setting Boundaries on Social Media
Session 15: You Can Manage Stress	Healthy Aging	Setting Boundaries by Learning to Say “No”
Session 16: Ways to Stay Motivated	Health Preparedness	Cognitive Restructuring
Session 17: Welcome to the Next Phase	Hair Care	Body Image and Self-Talk
Session 18: Food Preparation and Recipe Modification	Maternal Health and Menopause	Art as a Stress Reliever
Session 19: Healthy Eating – Taking It One Meal at a Time	Food Safety	Navigating Resources for Counseling and Therapy
Session 20: Healthy Eating with Variety and Balance	Breast Self-care and Mammograms	Self-Advocacy in Health Care
**Monthly Sessions**		
Session 21: Preventing Relapse	Dental Care	Guided Imagery
Session 22: Staying on Top of Physical Activity	Sleep Habits	Social Support
Session 23: Stepping Up to Physical Activity	Domestic Violence	Sleep Strategies
Session 24: Balance Your Thoughts for Maintenance	Vaccines	Distraction Techniques
Session 25: Handling Holidays, Vacations, and Special Events	Women’s Health Take-Home Points	Stress Management Take-Home Points
Session 26: Final Session: Look Back and Looking Forward	Women’s Health Take-Home Points	Stress Management Reflection

^a^Behavioral weight loss

The stress management strategies are evidence-based and were selected to address stressors that adult Black females with obesity had reported as culturally salient barriers to weight loss in our preliminary studies [37] (Table 2). Stress management training will include cognitive, behavioral, and relaxation coping strategies guided by Kreuter’s framework for appropriate cultural adaptation of behavioral interventions, which uses five strategies: *peripheral, evidential, linguistic, constituent-involving, and socio-cultural* [38] (Table 3). Cognitive strategies such as challenging dichotomous thinking and making positive self-statements that have been shown to reduce stress [39] will be taught as one approach. Participants will receive instruction in evidence-based behavioral strategies [40] including time management, financial management, and distraction techniques (e.g., listening to music) [40, 41], as well as relaxation techniques such as diaphragmatic breathing, progressive muscle relaxation, guided imagery, and massage [39, 41, 42]. These evidence-based techniques will be linked with needs, values, beliefs, and culture of the target population (e.g., collectivism, racial pride, religiosity/spirituality) to provide culturally relevant strategies for stress management among Black females with elevated stress. While we are primarily focused on culturally-targeted stress management strategies, some elements of the cultural targeting, e.g., racial- and gender-concordant interventionists, will naturally be imbedded into the BWL portion of the intervention. Participants will also receive a voucher for a monthly Swedish massage from licensed therapists at a local spa; the therapists will follow a standardized protocol to minimize technique variability across participants.

**Table 2:** Excerpt of results from formative assessments identifying stressors that are relevant to adult Black females and weight management

Sample Responses	Theme	Examples of Resulting Stress Management Enhancements
Q1. Taking care of everyone Q2. Child care as a single parent Q3. No time to eat right because I do for everyone else	Daily/Caregiver stress	-Benefits of massage -Time management -Social support/collectivism
Q1. Advancing in my career Q2. Job discrimination and being the only Black woman in the office	Work stress	-Challenging dichotomous thinking -Diaphragmatic breathing
Q1. Women don’t get the same chances as men Q2. I’m always worried about safety raising Black boys and being married to a Black man	Stigma, Discrimination, Bias	-Positive self-statements with pride in race and sex -Guided imagery
Q1. Paying for college as a single mother Q2. Not making as much money on jobs as men or white women Q3. Being healthy is expensive and hard for people with limited money	Financial stress	-Financial management and spiritual messages about provision
Q1. Trying to be in a good, happy relationship Q2. It’s hard for Black women to find a good Black man Q3. A lot of what I eat is just because of my husband	Relationship stress	-Distraction techniques -Meditation with focus on peace, spirituality, and pride

**Table 3:** Alignment of stress management intervention with Kreuter’s framework for cultural appropriateness in behavioral interventions

Categories for enhancing cultural appropriateness	Stress management enhancements	Examples
Peripheral	Racial, sex-specific, and cultural concordance	-Race- and sex-concordant interventionist; culturally concordant images in stress management materials
Evidential	Race- and sex-specific statistics on topic area	-Black women report higher chronic stress than white women and tend to use coping strategies that exude strength, which may negatively impact health
Linguistic	Use of dominant native language of target group	-Written and verbal stress management training will be presented in English; race- and sex-concordance of interventionists will increase the likelihood of culturally consistent meaning of language
Constituent-Involving	Identification of culturally relevant stressors and use of direct quotes from formative assessments in materials	- Job and race/sex-related stress: “I am mostly stressed out by my job. It is already challenging and I am the only black woman in the office” - Financial stress: “As a single parent, it’s hard putting kids through college”
Socio-Cultural	Incorporation of stress management strategies that are relevant to the values, beliefs, and culture of the targeted population, including religiosity/spirituality, racial pride, collectivism, and perception of time	-Meditation that includes elements of spirituality (prayer, scripture) -Positive self-statements that include

Participant implementation of the stress management strategies will be monitored by a daily autogenerated text messaging prompt that will ask “Did you use any of the stress management strategies today that you have learned as a part of the RESET Study?” If the response is “yes,” participants will receive another prompt instructing them to describe the stress management strategy that was used. This will allow not only insights into the preferential engagement in the different strategies introduced and shed light on the stress coping strategies perceived as most useful by Black women engaged in weight control efforts but will also provide an index of participant skill enactment and treatment fidelity.

### BWL-alone

BWL-Alone will meet in 60-min group sessions which will present the same DPP-based lifestyle change curriculum and will be augmented with a 20-min session on general women’s health topics (e.g., breast health, dental care) as an attention placebo to account for any impact that attention from the interventionist might produce (Table 1). The total session time will be approximately 80 min. The use of women’s health topics as an attention control has been successfully used before in the BWL literature [43] and in our pilot study [24]. For the 20-min, women’s health-focused sessions, the interventionist will review and summarize the current literature and guidelines from reputable publicly available sources (e.g., Centers for Disease Control; National Institutes of Health). Stress management will not be addressed in these women’s health topics to avoid contamination.

### Intervention materials

Participants will receive written materials for each session, sets of measuring cups and spoons, and a food scale to assist with self-monitoring. Written materials will be emailed weekly, and other supplies will be distributed at the baseline assessment. Participants will track their weight, dietary intake, and activity in MyFitnessPal, an app-based self-monitoring smartphone program. Participants will also receive automated weekly texts prompting them to report a time in the past week when they used strategies discussed in the sessions (either stress or women’s health). For example, a BWL-Stress participant may record a stressful event and the coping strategy that she implemented. A BWL-Alone participant may document conducting a self-breast exam. These records will be collected weekly to serve as adherence and engagement data in analyses comparing the two intervention conditions.

### Treatment Fidelity and staff training

All interventionists delivering the group-based sessions will receive initial training and then meet weekly with the study PI to review issues or concerns and ensure consistent delivery of the intervention. All treatment sessions will follow a structured outline of content that will also be reviewed at the weekly meeting preceding each treatment session. Additionally, a random sample of treatment sessions (10%) will be audio-recorded for evaluation by research staff (EE) to ensure treatment fidelity. Corrective feedback will be provided to interventionists as needed.

## Interventionists

Group-based treatment sessions will be led by Black female master’s level staff trained in health behavior/health promotion with extensive experience in BWL interventions. Since racial concordance of interventionists and participants is neither positively nor negatively associated with weight loss outcomes for Black participants [44], and Kreuter’s framework suggests that these types of “peripheral strategies,” similar to “surface tailoring” as introduced by Resnicow et al. [45] can enhance credibility and acceptance of a group, racially- concordant interventionists will be used. The BWL-Stress and BWL-Alone groups will be led by different interventionists to minimize the risk of across-group contamination. Interventionists will be similar in terms of training and experience and will be trained to employ a similar style to minimize the potential for interventionist effect.

### Changes to the protocol due to COVID-19

Due to the COVID-19 crisis, the proposed in-person intervention from the initial protocol design has been adapted for implementation via Zoom. Activities from the DPP curriculum (e.g., food demonstrations) have been modified for the online format, and the number of site visits has been reduced to assessment visits only. Weigh-ins will only be conducted at assessments (four times per cohort) instead of the proposed 26 in-person intervention sessions and four assessment visits. Participants will be asked to weigh themselves weekly and record their weights within the MyFitnessPal app, where they will also be asked to self-monitor their dietary intake and activity. Intervention materials (i.e., worksheets and information sheets) will be emailed to participants prior to the intervention session. Participants will be asked to print their own materials. To further reduce site visits, participants will be asked to mail their saliva kits and activity monitors to a member of the study team for analysis. Non-monetary incentive benchmarks will be aligned with assessment visits instead of being spaced out throughout the duration of the study. Participants will track their dietary intake and physical activity using the MyFitnessPal app instead of a food and activity diary. Lastly, to foster a positive group dynamic, interventionists will be trained to engage participants via Zoom (e.g., using polls and online whiteboards), and participants will receive Zoom backgrounds for the sessions. All adjustments have been discussed in detail among the investigative team and in consultation with other experts. The decisions were made to use approached that will minimize any impact on scientific rigor. Modifications have been approved by the Scientific Review Committee (SRC) and Institutional Review Board (IRB).

### Assessment design and outcome measures

Data will be collected at baseline and months 4, 6, and 12. All assessments will be conducted by trained research staff who will undergo regular protocol-driven training and monitoring to ensure rigorous and unbiased data collection. Calibrated digital monitoring devices will be used for clinical measures (e.g., weight, height, blood pressure, blood glucose) to minimize error or bias. All study assessments will be conducted at Moffitt Cancer Center.

#### Primary outcome: anthropometry

Height and weight will be measured following a standardized protocol using a SECA 2-in-1 scale and stadiometer. Both measurements will be performed with participants’ shoes removed and wearing light clothing. BMI will be calculated from measured height and weight. Our primary outcome is 6-month weight loss. We will also examine weight loss at 12 months for maintenance outcomes.

#### Secondary outcomes

##### Stress

Participants’ perception of psychological stress will be assessed using the PSS-10 and STRAIN. The PSS-10 measures the degree to which an individual perceives life events to be stressful. Sample items include: “In the past month, how often have you felt that you could not cope with all the things that you had to do?” and “How often have you felt that you were unable to control the important things in your life?” Responses are on a 5-point Likert sale (0 = never to 4 = very often). Four items (e.g., “felt confident about ability to handle personal problems”; “I felt things were going my way”) are positively worded and will be reverse coded for analysis. Higher total scores correspond to greater perceived stress. The PSS-10 has been validated in diverse populations and demonstrated to have good internal consistency (α = 0.77) in a previous study conducted by our group among a similar population [46]. STRAIN is an instrument that measures cumulative exposure to life stress, with questions on acute life events, chronic difficulties, and early life stressors [28]. STRAIN contains approximately 220 total questions, with automated omission of questions that are inappropriate for the respondent’s demographic characteristics. STRAIN is structured with close-ended core questions about 55 distinct stressors related to major life domains (e.g., health, education, work, finances, housing, living conditions, intimate relationships, friendships, crime, etc.) and follow-up questions regarding the severity, frequency, timing, and duration of each stressor. A set of 20 lifetime stressor scores will be created based on STRAIN responses. Objective stress will be measured by salivary cortisol via SalivaBio Oral Swab (Salimetrics, LLC, Carlsbad, CA, USA) at baseline and 6-months to correspond with the primary study outcome of weight loss at 6 months. Participants will be provided with a Salimetrics Swab Saliva Collection Kit that includes 9 swabs and tubes. Participants will be asked to collect saliva upon waking and at 30- and 60-min after waking for 3 consecutive days. Participants will be asked to store the samples in their home freezer until they return the samples to our research team after completion at each time point.

##### Waist circumference

Waist circumference will be measured using a Gulick constant-tension spring-loaded tape device on bare skin at the end of a normal expiration at the natural indentation between the 10th rib and the iliac crest to the nearest 0.5 cm.

##### Blood glucose

Blood glucose will be assessed from a blood sample collected via fingerstick and read by a calibrated monitor (Roche Diagnostics).

##### Blood pressure

Blood pressure will be measured using standard procedures at rest, in duplicate, using a WelchAllyn blood pressure monitor at each assessment time point. Measurements will be averaged at each time point.

##### Energy intake

Energy intake will be assessed by 24-h dietary recalls conducted using the National Cancer Institute’s Automated Self-Administered 24-Hour Recall tool (NCI ASA-24). The NCI ASA-24 is drawn from the USDA’s Automated Multiple-Pass Method which uses the “gold standard” multiple-pass methodology. This web-based program allows researchers to enter dietary recall data via a respondent portal that is linked to a researcher site where data are saved for future download. The NCI ASA-24 uses Computer-Assisted Self-Interviewing (CASI) methodology to guide the respondent through multiple steps of recall, including meal-based quick list, meal gap review, detail pass (including quantity consumed), forgotten foods, and final review. Data will be quantitated to assess energy and nutrient intake, and average daily caloric intake will be the parameter of analysis. Each assessment will include a weekday and weekend day to account for eating variations.

##### Physical activity (PA)

PA will be assessed via activity monitors (i.e., Fitbit Inspire 2) worn by participants for 7 days. Participants will be instructed to position the activity monitor on their non-dominant wrist and to wear the device throughout the day. A valid day of data will be considered at least 10 h of waking wear time. These data will be used to estimate total weekly minutes of moderate-to-vigorous intensity PA. Self-reported PA will also be assessed with the Paffenbarger Physical Activity Questionnaire (PPAQ). Activity monitors provide objective information about total levels of activity, while the PPAQ provides additional details about the type and schedule of activities.

##### Treatment adherence

Treatment adherence will be assessed by the number of sessions attended and the proportion of days when self-monitoring of dietary intake is completed [24, 30, 47, 48]. Research staff will review and record self-monitoring on the MyFitnessPal app to provide data on self-monitoring adherence.

##### Treatment burden

Behavioral burden of treatment will be assessed with a 15-item survey measuring the burden of treatment, including behaviors of self-monitoring, dietary changes, maintaining physical activity, and the impact of treatment on social relationships, which will be administered at visits at month 6 and month 12.

### Retention

Several strategies will be implemented to promote retention among study participants. In addition to receiving a CDC-recognized BWL program at no cost to them, participants will be provided with the results of their assessments, including BMI, waist circumference, blood glucose, blood pressure, and PSS-10 score. We will also send birthday and holiday cards to remain connected to participants. To further promote retention, we will provide participants with monetary incentives for completing each of three follow-up data collection visits, with an escalating scale of reimbursement to maximize the acquisition of follow-up data, including $50 at month 4, $100 at month 6, and $150 at month 12. Participants will also receive non-monetary incentives, i.e., merchandise with the study logo, such as a water bottle for perfect attendance by month 4 assessment, for achieving milestones. Finally, the research team will foster relationships and rapport with participants, which will positively impact adherence and retention. The research team will also have a weekly review of attendance to discuss participants at-risk for loss and discuss targeted strategies for retention (e.g., troubleshooting transportation challenges or scheduling conflicts) to proactively limit attrition. If a participant misses two consecutive intervention sessions, a member of the research staff will reach out to them directly to identify and discuss any barriers that may be able to be addressed.

### Data management and security

Data will be entered into a custom-designed database through REDCap [49]. Relational logic checks such as out-of-range values and internal consistencies will be developed and run periodically. Statistical reports will be generated to examine the total number of participants screened, consented, and randomized on study entry and follow-up, as well as a summary of demographic and baseline characteristics.

### Statistical analyses

For the primary aim which compares BWL-Stress and BWL-Alone on 6-month weight loss outcomes, weight change (baseline weight subtracted from 6-month weight), will be tested using linear models and adjusted for known prognostic measures for weight loss such as age [50] and baseline weight. Model diagnostics, such as assessing normality of residuals and homoskedasticity, will be performed. If any assumption is violated, appropriate transformations (e.g., log) or analyses (e.g., quantile regression) will be performed. Follow-up analyses will be conducted to examine weight loss at month 12.

The secondary aim examines change in perceived, self-reported psychological stress, and will specifically examine changes in PSS-10 and STRAIN scores and cortisol by intervention group at month 6. Similar to Aim 1, change scores will be created and treated as continuous, and adjusted linear models and baseline outcome measure will be performed with appropriate diagnostics for model assumptions. Stress will also be examined at month 12 using data from the PSS-10.

For Aim 3, which examines whether the relationship between the intervention and weight change is mediated by perceived stress, energy intake, and physical activity, we will determine whether the relationship between each intervention condition and weight loss is mediated by perceived stress (as measured by the PSS-10), caloric intake, or physical activity [51]. Each potential mediator will be evaluated independently using a counterfactual approach [52]. As we are interested in multiple mediators which likely are not independent, we will use inverse odds ratio weighting (IOWR) to estimate the natural direct effect of BWL-Stress and BWL-Alone [53, 54]. This method first estimates weights by using logistic regression (to predict the dichotomous exposure variable) containing all mediators and confounders and creates an odds ratio for each participant. Next, the odds ratios (OR) are inverted (f (OR_i_) = OR_i_^− 1^) for each participant to create an IORW weight with standard BWL participants having weight 1. Direct effects of the intervention are estimated using weighted linear regression models including confounders and no mediators using IORW weights. The total effect of the intervention on weight loss is then estimated using an unweighted linear regression with the same confounders as the weighted analysis. The indirect effect is then estimated by subtracting the direct effect from the total effect. Confidence intervals for the effects are created using bootstrap methods. All analyses will be intention-to-treat. Baseline observation carried forward, which conservatively estimates a return to pre-treatment weight, will be used for participants without follow-up outcome measures for the primary outcome of weight. For participants missing data other than the primary outcome, we will perform multiple imputation as appropriate, as complete case analyses are known to be biased.

Missing data will be handled using multiple imputation by chained equations (MICE) with auxiliary variables identified using procedures from Enders [55]. Estimates from different imputed datasets will be combined using Rubin’s rules, and these will be integrated with inverse weighting mediation measures based on Stata code from previous similar work [55]. Multiple imputation is superior to complete case analysis if the missing complete at random assumption is violated. The likelihood of meeting the missing at random assumption will be bolstered with the use of auxiliary variables.

### Ethics

The study received scientific approval from the Moffitt Cancer Center behavioral science scientific review committee, and ethical approval from the Advarra Institutional Review Board (IRB) (Pro00053438). Written informed consent will be obtained from all participants following procedures approved by the IRB. Further, study recruitment, intervention delivery, and retention will be monitored by a Data Safety Officer at biannual intervals.

## Discussion

Obesity, a risk factor for many chronic diseases, is disproportionately represented among minority populations, with the highest prevalence among Black females [4]. Over half of adult Black females in the United States have obesity [4] and Black females lose less weight than White females when engaging in obesity treatment, regardless of the treatment modality [56]. Black females also have a higher prevalence of obesity-associated diseases, including type 2 diabetes, compared with White females [57]. While it is clear that creating an energy deficit through decreased intake or increased expenditure is required to promote weight loss, research shows that energy intake and expenditure are associated with many biological, psychosocial, environmental and behavioral factors that may vary across population subgroups [58]. Thus, obesity treatment is not a “one size fits all” regimen, and targeted approaches may lead to improved outcomes, especially among high-risk populations.

This study will examine whether culturally-salient stress management strategies can improve weight loss outcomes for Black females with obesity and elevated stress and will identify the mechanisms that link stress management to improved weight loss. Findings from this study may provide tools to improve weight loss outcomes for a high-risk population that has demonstrated only modest responses to other obesity treatment options. Findings may also provide a model to address other chronic diseases such as diabetes, cancer, and cardiovascular disease. If shown to be effective and clinically meaningful, the novel intervention examined in this study will also support future dissemination and implementation studies to integrate this approach into current community-based and clinical obesity treatment programs.

Completion of this proposal will determine if systematically addressing stress in BWL programs can improve outcomes for adult Black females with elevated stress and identify the underlying behavioral mechanisms. If the novel intervention is effective, study findings can be used to improve obesity treatment for the segment of the U.S. population with the greatest burden of obesity and may be adapted for other populations.

## Data Availability

Data sharing is not applicable to this article as no datasets were generated or analyzed during the current study.

## Abbreviations

BWL: Behavioral weight loss
BMI: Body mass index
CAB: Community Advisory Board
CASI: Computer-Assisted Self-Interviewing
CDC: Centers for Disease Control
DPP: Diabetes Prevention Program
MICE: Multiple imputation by chained eqs.
NCI ASA-24: National Cancer Institute’s Automated Self-Administered 24-Hour Recall
OR: Odds ratio
PA: Physical activity
PPAQ: Paffenbarger Physical Activity Questionnaire
PSS-10: Perceived Stress Scale
STRAIN: Stress and Adversity Inventory
